# The Medical Council and the Curriculum

**Published:** 1900-12-15

**Authors:** 


### The Hospital

A Journal of

The Medical Sciences and Hospital Administration.

Vol. XXIX.—No. 742.] Edited by Sir Henry Burdett, K.C.B., and Solomon C. Smith, M.D., M.R.C.P. [December 15, 1900.

### THE MEDICAL COUNCIL AND THE CURRICULUM.

The state of tension which has come into existence between the General Medical Council and some of the licensing bodies, whose representatives are among its members, with reference to the first year of what is intended to be medical study is on all accounts much to be regretted. Briefly stated, the question at issue is whether instruction in physics, chemistry, and biology, not given at a university or school of medicine, but at an ordinary boys' school during the last year of pupilage there, is sufficient to exalt this last year of "schooling" into the position of the first year of the five over which it is the desire of the Council that the curriculum of distinctively medical education should now extend, thus leaving only four years, as heretofore, to be spent at a medical school properly so called. The Conjoint Board, formed in London by the Royal Colleges of Physicians and of Surgeons, maintains the affirmative of this proposition, and is prepared to set the Medical Council at defiance in so maintaining it; while the Royal College of Surgeons of Ireland has announced that it will not be bound by any restrictions which the English Colleges have cast aside. A memorandum prepared by Mr. Bryant, the representative of the Royal College of Surgeons of England on the Council, practically admits that the whole question is one of the pecuniary interests of the licensing bodies, as these are affected by competition, and states that the London Colleges were "unable to maintain" the "advanced position" which at one time they took up, and that they therefore "adopted," although with regret, the "ways" of the other colleges and universities which count, as part of the five years' curriculum, the time spent in the acquisition of physics, chemistry, and biology when taken either at a medical school or at a "*recognised teaching institution.*" The words which we have italicised mean any common school for children, it may be a Board school, or an "Evening Continuation" thereof, which the authorities of any licensing body may choose to consider good enough for "recognition."

The chief purpose for which the Medical Council was established, and, indeed, the *raison d'être* of its existence, was said in the House of Commons, by the introducer of the Bill which became the Medical Act of 1858, to be that it "would fill the functions of visitors in the existing licensing bodies, and would ensure efficiency of *teaching* and examination." It was felt, even then, that absolutely unrestricted competition among licensing bodies would not be conducive to the maintenance of a high standard of medical education and qualification, and the Council was formed for the protection of the public in this respect. It does not appear, however, that it was provided with sufficient powers for the proper performance of its duties. It has no control over teaching; and all it can do with regard to examination is to represent to the Privy Council that a given examination is, in its opinion, "insufficient," the Privy Council itself being then called upon to inquire into and determine the question. The Council instituted a "registration" of medical students, from which the commencement of the required curriculum has hitherto been dated, and it now threatens to refuse registration to students who are still in the position of common schoolboys. The licensing bodies assert that the threat is an empty one, that registration is a superfluous formality, that they can and will examine and license candidates who have never been registered at all, that the Council has no authority over *teaching*, and that its right of complaint to the Privy Council, on the score of the insufficiency of an examination, is strictly limited to the "final" or qualifying examination in "medicine, surgery, and midwifery," and does not extend to any earlier or intermediate ones. Moreover, they take the high ground of asserting that the full responsibility for the conduct of medical education and examination has been committed to them by Charters and by Acts of Parliament, and that they would be abandoning their rights and duties if they were to permit the interference of the Council in such matters. The Council, nevertheless, by a large majority, has determined to stick to its guns, and "to adhere to its resolution with regard to the registration of medical students," which, therefore, will not be permitted until they have actually commenced attendance "at a university, school of medicine, or scientific institution approved by the Council."

If the question which has thus been raised should come before the Courts, its decision would depend, of course, upon the precise interpretation which Her Majesty's Judges might be pleased to attach to the conglomerate of contradictory blunders known commonly as an Act of Parliament. Before the tribunal of enlightened opinion it presents itself under a somewhat different aspect. Taking chemistry alone, it is inconceivable that the amount of this science taught to a schoolboy would be sufficient either to answer any useful purpose as an instrument of mental training, or to enable him to understand the new lights which the work of chemists is beginning to throw upon some of the most abstruse problems connected with nutrition and with disease. We deeply regret to see the Royal Colleges ranging themselves on the side of smattering, and in opposition to the constantly increasing educational requirements of the day. Pasteur explained the absence of great men in France, at the time of her prostration before Germany, by the national neglect of education in the exact sciences; and added: "Pour prendre notre revanche il faut tout refaire du haut en bas, *du haut surtout.*" If the Royal Colleges prefer sciolism to science, it will be necessary, before long, to supersede their licenses by a State Examination, controlled and directed in the interests of medical efficiency and of public safety.

